# Prognostic Impact of Canonical TGF-β Signaling in Urothelial Bladder Cancer

**DOI:** 10.3390/medicina55060302

**Published:** 2019-06-24

**Authors:** Slavica Stojnev, Miljan Krstić, Jovana Čukuranović Kokoris, Irena Conić, Ivan Petković, Sonja Ilić, Jelena Milosević-Stevanović, Ljubinka Janković Veličković

**Affiliations:** 1 Department of Pathology, Faculty of Medicine, University of Niš, 18000 Niš, Serbia; krstic.miljan@gmail.com (M.K.); dravel@open.telekom.rs (Lj.J.V.); 2 Center for Pathology, Clinical Center Niš, 18000 Niš, Serbia; 3 Department of Anatomy, Faculty of Medicine, University of Niš, 18000 Niš, Serbia; jovana.c85@gmail.com; 4 Department of Oncology, Faculty of Medicine, University of Niš, 18000 Niš, Serbia; irenaconic@yahoo.com (I.C.); ivan76.unsu@yahoo.com (I.P.); 5 Clinic of Oncology, Clinical Center Niš, 18000 Niš, Serbia; 6 Department of Physiology, Faculty of Medicine, University of Niš, 18000 Niš, Serbia; sonjaili@yahoo.com; 7 Department of Gynecology and Obstetrics, Faculty of Medicine, University of Niš, 18000 Niš, Serbia; jelamilostev@gmail.com; 8 Clinic of Gynecology and Obstetrics, Clinical Center Niš, 18000 Niš, Serbia

**Keywords:** urothelial bladder cancer, TGF-β, Smad2, Smad4, immunohistochemistry, prognosis

## Abstract

*Background and objectives*: Dysregulation of TGF-β signaling plays multiple roles in cancer development and progression. In the canonical TGF-β pathway, TGF-β regulates the expression of hundreds of target genes via interaction with Smads, signal transducers and transcriptional modulators. We evaluated the association of TGF-β1, Smad2, and Smad4, the key components of canonical TGFβ pathway, with clinicopathologic characteristics of urothelial bladder cancer, and assessed their prognostic value in prediction of patients’ outcome. *Materials and Methods*: Immunohistochemical analysis of TGF-β1, Smad2, and Smad4 expression was performed on 404 urothelial bladder cancer samples, incorporated in tissue microarrays. Expression status was correlated with clinicopathological and follow-up data. The median follow-up was 61 months. *Results*: High expression of TGF-β1, Smad2, and Smad4 was detected in 68.1%, 31.7% and 45.2% of the tumors, respectively. TGF-β1 overexpression was significantly associated with high tumor grade, and advanced pathologic stage (*p* < 0.001, respectively). Conversely, high Smad2 and Smad4 expression was linked to low tumor grade (*p* = 0,003, *p* = 0.048, respectively), and low tumor stage (*p* < 0.001, *p* = 0.003, respectively). Smad2 showed an inverse correlation with variant morphology and divergent differentiation of urothelial tumors (*p* = 0.014). High TGF-β1 correlated directly, while Smad2 and Smad4 correlated inversely to cancer-specific death (*p* = 0.043, *p* = 0.003, and *p* = 0.022, respectively). There was a strong relationship between Smad2 and Smad4 expression (*p* < 0.001). Survival analyses showed that high Smad2 and Smad4 expression was associated with longer overall survival (*p* = 0.003, *p* = 0.034, respectively), while in multivariate regression analysis TGF-β1 manifested as an independent predictor of poor outcome. *Conclusions*: Unraveling the complex roles and significance of TGF-β signaling in urothelial bladder cancer might have important implications for therapy of this disease. Assessment of TGF-β pathway status in patients with urothelial bladder cancer may provide useful prognostic information, and identify patients that could have the most benefit from therapy targeting TGF-β signaling cascade.

## 1. Introduction

Bladder cancer is a common malignancy of urinary tract, the ninth most frequent cancer worldwide, and the 13th deadliest, with the highest mortality rates in Eastern and Sothern Europe, and in the Baltic countries [1,2]. This neoplasm represents a remarkable burden for a healthcare system, because the patients are condemned to a life-long surveillance with cystoscopic controls, and often require treatment of recurrent disease [1]. Urothelial carcinoma accounts for more than 90% of bladder cancer. Recent studies have recognized that urothelial bladder cancer (UBC) is a heterogeneous pathologic entity, with multiple molecular subtypes, that surpass the traditional classification of the disease based on tumor stage and histological grade. New insights in complex molecular landscape and alterations in numerous signaling pathways in UBC have opened new possibilities in prognosis, follow-up, and personalized treatment of the patients [3,4].

Transforming growth factor beta (TGF-β) family of proteins includes multifunctional molecules that regulate cell proliferation, differentiation, apoptosis, interaction with the microenvironment, and immune reactivity [5,6]. Dysregulation of TGF-β signaling pathway plays multiple roles in cancer development and progression. TGF-β1, the foremost member of the family, is a notoriously pleiotropic cytokine, which may evince tumor-suppressor properties, and also mediate numerous processes essential for cancer advancement, including cell invasion and metastasis [5]. Moreover, TGF-β is the key inducer and regulator of epithelial–to–mesenchymal transition (EMT), a fundamental, physiological process during morphogenesis, by which cell phenotype is converted from polarized epithelial to mesenchymal, associated with migration capacity and stem features. EMT is critical for cancer progression and metastatic spread [7,8], where it reflects impressive phenotypic plasticity of the cancer cell. The major importance of TGF-β–triggered EMT has recently been described in invasion and dissemination of UBC [9,10].

In the canonical TGF-β pathway, TGF-β regulates the expression of hundreds of target genes via interaction with Smads, signal transducers and transcriptional modulators, which activate or repress various transcription factors [11]. Binding of active TGF-β1 to type I and type II receptors leads to signal propagation through phosphorylation of Smad proteins. Initially, receptor activation phosphorylates receptor substrate Smads, Smad2 and Smad3, which form a complex with Smad4, and thereafter translocate to the nucleus. This complex binds to specific gene targets, together with the assistance of DNA-binding cofactors, and modifies the expression of many genes. Classical TGF-β activated Smad signaling can exert both pro- and anti-tumor activities in oncogenesis, in a context-dependent manner [11,12].

Genetic variations of several components of the TGF-β canonical pathway have been significantly linked to bladder cancer risk [13,14]. Mutations of *SMAD2* are infrequent in cancer, they are found in a small fraction of colorectal carcinoma, while, on the other hand, *SMAD4/DPC4* is considered an important tumor-suppressor gene [15,16]. The loss of heterozygosity of chromosomal locus 18q21—which contains *SMAD2* and *SMAD4* genes—is a common event in pancreatic and colorectal cancer. The alterations in Smad2 and Smad4 expression have been recently described in UBC [16,17], but their association with clinicopathologic features and their clinical significance have not been elucidated so far.

The aim of this study is to evaluate the association of TGF-β1, Smad2, and Smad4, the key components of canonical TGFβ pathway, with clinicopathologic characteristics of UBC, and to assess their prognostic value in prediction of patients’ outcome.

## 2. Materials and Methods

### 2.1. Patients and Histopathologic Analysis

The present study comprised cancer tissue samples of 404 patients with urothelial bladder cancer who underwent transurethral resection of bladder tumor between January 2007 and December 2012 in Clinic of Urology, Clinical Center Nis, Serbia. All cases were diagnosed at the Center for Pathology, Clinical Center Nis, Serbia. The average patients’ age was 66.6 ± 9.8 years, with the predominance of male patients compared to female (77.2% vs 22.8%). Only patients whose updated and detailed medical records were available were included in the study. Gathered data encompassed survival time, disease-free survival, and recurrence, as well as the cause of death. Cancer-specific death was defined as death caused by bladder cancer, and it did not include mortality caused by other neoplastic or non-neoplastic disease. The median follow-up was 61 months (25 to 125 months). Follow-up time was expressed as the number of months, from diagnostic transurethral resection to the last control visit or death. The study was approved by the Ethics Committee of the Faculty of Medicine, University of Nis, Serbia (Decision No 12-1250/8; approval date: 6 February 2018).

Formalin-fixed paraffin-embedded cancer tissue sections stained with hematoxylin and eosin were used for diagnosis and assessment of pathologic parameters. Pathologic classification of urothelial bladder cancer was performed according to the 2016 World Health Organization classification [18], and the Eighth edition of UICC TNM Classification was used for pathologic staging [19].

### 2.2. Immunohistochemical Analysis

For the purpose of immunohistochemical analysis tissue microarray blocks (TMAs) were constructed using the manual tissue arrayer. Every tumor was represented in a recipient TMA block by two core cancer tissue samples with a diameter of 2 mm. In addition, every TMA block included samples of the normal bladder mucosa.

Immunohistochemical analysis was performed using the primary antibodies to TGF-β1 (clone TGFB17, Leica Biosystems Newcastle, 1:40 dilution), Smad2 (ab63576, Abcam, 1:100 dilution), and Smad4 (ab236321, Abcam, 1:100 dilution). In brief, four micrometer thick TMA sections were deparaffinized in xylene and rehydrated in a graded ethanol series. Heat–induced antigen retrieval procedure was performed in a microwave oven for 20 min with citrate buffer (pH 6.0). After endogenous peroxidase activity block and thorough rinsing with phosphate buffered saline, the slides were incubated with the primary antibody for one hour at room temperature. Immunostaining procedures included appropriate controls. A standard immunoperoxidase detection system was applied, and diaminobenzidine (DAB) was used as a chromogen for the visualization of reaction. Slides were counterstained with Mayer’s hematoxylin, dehydrated in ethanol and xylene, and mounted with DPX.

Immunoexpression of TGF-β1 and Smads was considered high if at least 50% of cancer cells were stained with intermediate or strong brown color intensity, as described elsewhere [20]. TGF-β1 displayed cytoplasmic and/or membranous staining pattern, while Smad2 and Smad4 showed nuclear and cytoplasmic staining, with predominantly moderate intensity. Pathological analysis and interpretation of immunohistochemical stains were performed on the light microscope (Olympus BX43, Olympus Corporation, Tokyo, Japan). Digital photographs were acquired using the imaging system (Olympus cellSens platform standard, Olympus Corporation, Tokyo, Japan).

### 2.3. Statistical Analyses

All data analyses were processed using the Statistical Package for Social Sciences, version 24.0 statistical software (SPSS, Chicago, IL). The association of markers’ expression and clinicopathologic features were tested by the χ2 test. The differences between Kaplan–Meier survival curves were tested for statistical significance by the log-rank test. Cox regression analysis with the enter method was performed to explore the relationship between the survival of the patients and explanatory variables. This analysis included categorical independent variables: histological grade, tumor stage (reference category was pTa, non-invasive tumor stage), variant differentiation, carcinoma in situ, and expression of investigated markers. A *p*-value of 0.05 or less was considered indicative of a statistically significant difference.

## 3. Results

### 3.1. Expression of TGF-β1, Smad2, and Smad4 in Relation to Clinicopathologic Characteristics

**Figure 1:**
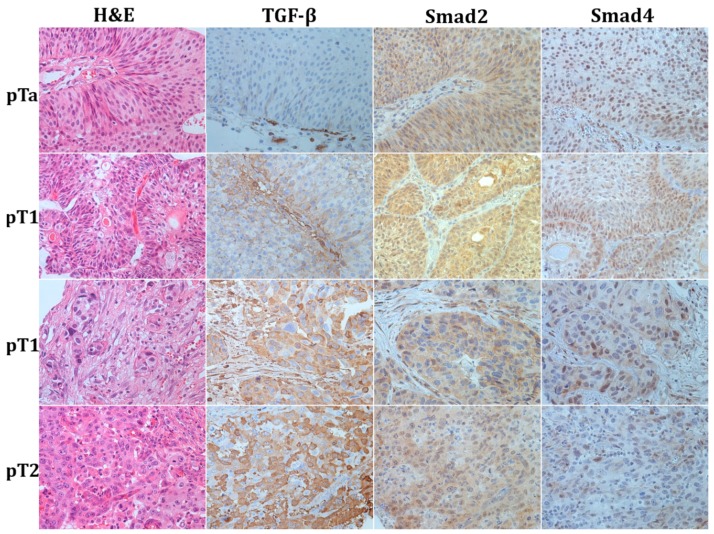
Representative photomicrographs of haematoxylin–eosin stain and immunohistochemical staining to TGF-β1, Smad2, and Smad4 in urothelial bladder cancer; first row—papillary non-invasive low grade tumor (pTa); second row—superficially invasive low grade tumor (pT1); third row—superficially invasive high-grade tumor (pT1); fourth row—muscle-invasive urothelial bladder cancer (pT2). Original magnification ×400.

High expression of TGF-β1 was noted in 68.1% of tumors, predominantly as cytoplasmic staining, and less frequently in a pattern of perimembranous or linear membranous staining (Figure 1).

TGF-β1 overexpression was significantly associated with high tumor grade, and advanced pathologic stage (*p* < 0.001, respectively). The majority of urothelial tumors with high TGF-β1 expression were classified as high–grade neoplasms (70.5%). The proportion of TGF-β1–high tumors rose with the advancing stage: 45.4% (44/97) of non-invasive tumors, 73.8% (144/195) of early invasive and 77.7% (87/112) of muscle-invasive carcinoma overexpressed TGF-β1, with a highly significant difference between non-invasive and infiltrating urothelial carcinoma. Moreover, TGF-β1 expression was linked to female gender (*p* = 0.038), 76.1% of women had TGF-β1–high tumors versus 65.7% of men. High expression of Smad2 and Smad4 was observed in 31.7%, and 45.2% of tumors, respectively. High Smad2 and Smad4 nuclear expression with or without cytoplasmic staining in urothelial cancer (Figure 1) was associated with low tumor grade (*p* = 0.003, *p =* 0.048, respectively). Immunoreactivity to Smad2 was found in the majority of tumor cells in 40.5% (60/148) low grade, and in only 26.6% (68/256) high-grade neoplasms. Decrease or loss of Smad2 and Smad4 was significantly associated with advanced tumor stage (*p* < 0.001, *p* = 0.003, respectively). Smad2 immunoreactivity showed an inverse correlation with the increasing stage: high Smad2 was found in 44.3% (43/97) of pTa, 36.4% (71/195) pT1, and only 12.5% (14/112) of pT2 tumors. Smad4 expression also decreased with tumor progression. High Smad4 was found in 53.1% (51/96) pTa, 49.0% (95/194) pT1, and 31.8% (35/110) pT2 cancers. Moreover, Smad2 showed a strong inverse correlation with variant morphology and divergent differentiation of urothelial tumors (*p* = 0.014). Tumors with classic histomorphology more often expressed Smad2. Associations of TGF-β1 and Smad2 and Smad4 tumor status and clinicopathologic features are shown in Table 1.

At the designated endpoint of the follow-up period, 191 (47.3%) patients had died, and cause of death was declared as cancer-specific in 34.2% of patients. Overexpression of TGF-β1 directly correlated to cancer-specific death (*p* = 0.043). Conversely, Smad2 and Smad4 showed inverse correlation to cancer-specific death (*p* = 0.003, and *p* = 0.022, respectively). Loss of Smad2 and Smad4 indicated death outcome in cancer patients. There was no significant association between any of the markers and tumor relapse. In terms of treatment, patients with TGF-β1–positive tumors had a higher probability to be treated with systemic chemotherapy or radiotherapy (*p* = 0.017), and a significantly lower chance of receiving only immunotherapy with Bacillus Calmette–Guérin (BCG) vaccine. High Smad2 expression indicated a high probability of further treatment with intravesical instillation of BCG vaccine (*p* = 0.007), while its loss correlated with radical cystectomy (*p* = 0.004).

The analysis of markers’ expression correlation indicated a strong relationship between Smad2 and Smad4 expression (*p* < 0.001), while TGF-β1 did not correlate significantly with either of its downstream signaling molecules (*p* = 0.202, and *p* = 0.289 for Smad2 and Smad4, respectively). Eighty–one tumor (20%) showed uniform expression of Smad2 and Smad4, while 172 (42.57%) tumors were scored as simultaneously negative/low for the expression of both markers.

### 3.2. Association of TGF-β1and Smads Expression and Overall and Recurrence-Free Survival

**Figure 2:**
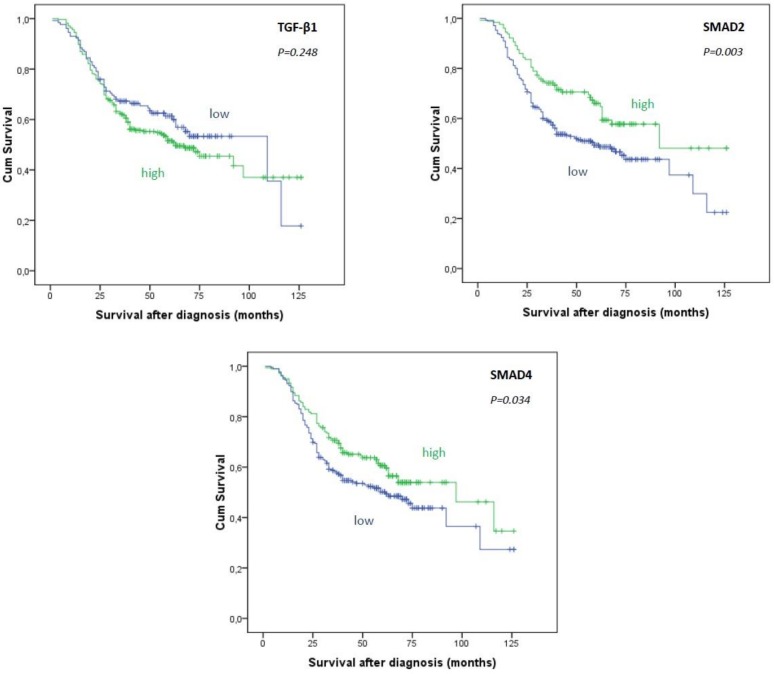
Kaplan–Meier survival curves showing overall survival in 404 patients with urothelial bladder cancer in relation to expression of TGF-β1, Smad2 and Smad4 in cancer cells. Log Rank (Mantel–Cox) test.

The mean survival time after diagnosis was 73.1 ± 2.9 months (mean ± SE) and the median survival was 73 months. The results of Kaplan–Meier survival analysis are shown in Figure 2.

Patients with TGF-β1–high tumors had shorter means and medians of survival time than patients with TGF-β1–low tumors (mean ± SE (median): 71.5 ± 3.5 (63 months) for TGF-β1–high vs 76.9 ± 4.7 (109 months) for TGF-β1–low tumors). However, this difference was not statistically significant (*p* = 0.248). Survival analyses showed that high Smad2 expression was associated with longer overall survival (*p* = 0.003). High expression of Smad4 was also linked to better prognosis (*p* = 0.034).

The mean recurrence-free survival in the investigated group was 67.6 months. Immunohistochemical expression of TGF-β1 did not correlate significantly with the recurrence-free survival (*p* = 0.681). In addition, Smad2 and Smad4 did not show significant association with recurrence-free survival (*p* = 0.950, *p* = 0.286, respectively).

Cox regression analysis indicated tumor grade, invasive pathologic stages pT1 and pT2, and TGF-β1 expression as independent predictors of patients’ survival (*p* < 0.001, *p* < 0.001, *p* = 0.041, respectively) (Table 2). High immunohistochemical expression of TGF-β1 in cancer cells was significantly associated with worse overall survival.

## 4. Discussion

In the era of personalized medicine and targeted therapy, well established prognostic parameters such as pathological stage and grade are not sufficient for accurate prognosis, selection of the best treatment modality, and prediction of tumor response to therapy. Molecular markers are very helpful in stratifying the patients with seemingly very similar tumors, and in identifying the patients who might benefit from adjuvant therapy [21]. Molecular genetics of bladder cancer has been extensively studied, but unfortunately there has not been any significant translation into progress of clinical management [8,22].

Neoplastic transformation is inherently associated with the disruption of TGF-β signaling, where TGF-β loses its tumor-suppressor capacities and transforms into pathologic signaling cascade that promotes carcinogenesis and ameliorates tumor growth. TGF-β1 strongly supports immune tolerance, thus tumors may employ high TGF-β1 production as an efficacious mechanism to elude immune response. In the setting of neoplastic disease TGF-β is involved in tumor invasion, dissemination of cancer cells and metastasis [5]. Alterations of TGF-β signaling result in production of cytokines that support tumor growth and spread, and in modification of microenvironment that stimulates EMT. It has been found that muscle-invasive bladder cancer highly expresses molecular markers associated with EMT [7,8,9]. Several other studies emphasized the importance of EMT for the pathogenesis of UBC, where TGF-β/Smad pathway was marked as the main inducer of EMT, and its ablation lead to inhibition of dissemination propensities [9,10]. In addition, a recent study indicated the key role of TGF-β in induction of cancer cell invasion in bladder cancer and identified TGF-β as the crucial motility factor in cancer cell migration [23].

One of the first studies that investigated the expression of TGF-β in 51 bladder cancer using a polymerase chain-reaction-based method suggested that the expression of TGF-β1 is significantly higher in cancer tissue than in normal bladder mucosa [24]. TGF-β1 expression was higher in low and intermediate grade tumors than in high-grade tumors, and in superficial tumors compared to muscle-invasive tumors. The authors concluded that levels of TGF-β1 are uniformly low in high-grade and invasive bladder cancer, which is in a collision with the results obtained in the present study. We observed that muscle-invasive and high-grade tumors showed unequivocally the highest TGF-β1 activity. However, a significant proportion of superficial papillary tumors also showed TGF-β1 overexpression. This may suggest that in various phases of UBC development TGF-β1 may impart different roles, often completely opposite ones, suppressive in early stages, and supportive in advanced stages of tumor progression. In addition, TGF-β1 was higher in carcinomas with squamous elements but there were only four of these tumors in the study group [24]. Our study included 45 tumors with squamous differentiation, but we found no correlation of TGF-β1 with divergent differentiation. However, high Smad2 expression significantly correlated with the classic tumor morphology and the absence of variant morphological features or divergent differentiation.

In the immunohistochemical study of TGF-β1, and its receptors I and II which comprised 80 UBC, high TGF-β1 expression was found in 64% of the cases, similar to our results [20]. We found that high TGF-β1 was strongly connected to the invasive tumor stage, which is also in accordance with previous results [20]. Moreover, overexpression of TGF-β1 was found to be associated with the loss of receptors I and II, which activation leads to signal propagation in the canonical TGF-β pathway. Although TGF-β1 signaling is pursued linearly via Smads in the canonical TGF-β pathway, we did not find a significant correlation between TGF-β1 and Smad2 and Smad4 expression. This is probably due to numerous modifying cofactors included in the signal transmission. On the other hand, Smad2 and Smad4 showed significant mutual correlation, which may reflect the joint action of these molecules. Namely, activated Smad2 forms a complex with Smad4 and translocates to nucleus to bind to specific but extensive gene targets, and this process recruits multiple activators, repressors, and chromatin remodeling molecules. This underlies the capability of TGF-β signal to regulate a multitude of functionally diverse target genes at once [5].

Not only the defect of TGF-β1, but also deletion of *SMAD2* and *SMAD4* genes in a murine model, renders animals more likely to spontaneously develop neoplastic growth [6]. A recent study has found that phosphorylated Smad2/3proteins are overexpressed in bladder carcinoma compared to adjacent normal tissue [17]. No significant correlation between high p-Smad2/3and clinicopathologic features of the patients was found. Another study suggested that the highest levels of phosphorylated Smad2 were present in high-grade urothelial carcinoma and were associated with increased recurrence rate [23]. However, our study did not show a significant relationship between the immunoexpression of any of the investigated proteins and disease recurrence.

Smad4, one of the key mediators of TGF-β signaling, is recognized as global tumor suppressor [25]. Juvenile polyposis syndrome, the appearance of multiple hamartomatous polyps in the gastrointestinal tract associated with the increased risk of adenocarcinoma, is caused by mutations in *SMAD4* gene [26]. Inactivation of *SMAD4* by mutation or deletion is well described as an important and common event in the development of pancreatic and colorectal carcinoma, and less frequently is observed in cancers of numerous other localizations, including urinary bladder [16,25]. In a study that comprised 34 urothelial carcinomas of the renal pelvis and ureter Smad4 expression was detected in 17.6% of the cases [27], which is a smaller percentage than in present study (45.2%). This difference may be attributed to several factors: use of different antibodies, specific localization of the tumors (upper vs lower urothelium), a small number of cases in the former study. Smad4 staining was observed in both, cytoplasm and nucleus of urothelial carcinoma. There is insufficient data about the significance of Smad4 staining pattern. In this study, exclusively cytoplasmic staining pattern alone, which was only seldom observed, was not significantly associated with tumor grade and stage. Usually, cytoplasmic staining was noted only as addition to nuclear immunoreactivity (Figure 1). This is in accordance with the notion that Smad4 exerts its DNA-binding properties within the nucleus, after translocation from the cytoplasm. For the first time in the analysis of Smad4 immunohistochemical expression in UBC, our results suggested that there is a significant relationship between Smad4 and tumor grade and stage. In addition, Smad4 staining of the majority of cancer cells significantly correlated to cancer-specific death. High Smad4, as well as Smad2 staining, indicates less aggressive tumors and longer cancer-specific survival.

The majority of patients with bladder cancer are diagnosed with non-muscle-invasive tumors. Most of these patients are treated with transurethral resection of the bladder tumor (TURBT), with or without the following intravesical immunotherapy with BCG or intravesical chemotherapy. A significant proportion of patients with muscle-invasive disease undergo cystectomy (about 40%), which may be followed by chemotherapy and/or radiotherapy. Around half of the patients with pT2 tumors are treated with TURBT followed by combined chemotherapy and radiation. Patients with metastatic disease at diagnosis receive cisplatin–based chemotherapy as a first line of treatment [28,29]. In our study, the expression of TGF-β1 in a tumor tissue was a strong indicator that the patient will be treated with chemotherapy and/or radiotherapy. Conversely, high Smad2 expression indicated further treatment with intravesical immunotherapy with BCG vaccine. The absence or low expression of Smad2 was strongly associated with radical surgical treatment.

To our knowledge, this is the largest study of prognostic value of TGF-β signaling molecules in urothelial bladder cancer. Our study suggests that overexpression of TGF-β1 is significantly associated with cancer–specific death. This is in agreement with the previous study that recognized TGF-β1 as an independent predictor of disease progression in patients with bladder cancer treated with radical cystectomy [20]. Expression of Smad2 and Smad4 predicts better prognosis and longer patients’ survival after diagnostic TURBT.

Several limitations of this study should be taken into account. One limitation is linked to the reproducibility and reliability of the immunohistochemical method. We used tissue microarrays and three pathologists independently reviewed the slides in an attempt to make the optimal assessment of staining. There is also limitation inherent to any retrospective study. However, this is the largest study sample of UBC assessed for the prognostic impact of canonical TGF-β pathway components. Further validation studies are warranted.

## 5. Conclusions

In conclusion, overexpression of TGF-β1 is common in urothelial bladder cancer, and it significantly correlates to invasive disease and high histological grade of the tumors. High TGF-β1 is associated with cancer–specific death, and represents the independent predictor of adverse prognosis. The immunohistochemical expression of downstream signaling proteins Smad2 and Smad4 is, on the other hand, associated with low grade tumors, superficial disease, and better overall survival of the patients. TGF-β actions are highly context-dependent, thus elucidating the role and significance of TGF-β signaling in cancer is quite a challenging task. Unraveling the complex roles and clinical significance of TGF-β in urothelial cancer will have important implications for therapy of this disease. Targeting TGF-β signaling is a promising course in the treatment of advanced and metastatic bladder cancer [30]. Assessment of TGF-β pathway status in patients with UBC may provide useful prognostic information, and identify patients that could have the most benefit from therapy targeting TGF-βsignaling cascade.

## Figures and Tables

**Table 1:** Association of TGF-β1, Smad2, and Smad4 expression with clinicopathologic features of bladder cancer.

		TGF-β1		Smad2		Smad4	
Characteristics		High	*p* value	High	*p* value	High	*p* value
Total (*n* (%))	404 (100)	275 (68.1)		128 (31.7)		181 (45.2)	
Gender							
Female	92 (22.8)	70 (25.5)	0.038	27 (21.1)	0.340	45 (24.9)	0.246
Male	312 (77.2)	205 (74.5)		101 (78.9)		136 (75.1)	
Tumor grade							
Low	148 (36.6)	81 (29.5)	<0.001	60 (46.9)	0.003	75 (41.4)	0.048
High	256 (63.4)	194 (70.5)		68 (53.1)		106 (58.6)	
Pathologic stage							
pTa	97 (24.0)	44 (16.0)	<0.001	43 (33.6)	<0.001	51 (28.2)	0.003
pT1	195 (48.3)	144 (52.4)		71 (55.5)		95 (52.5)	
pT2	112 (27.7)	87 (31.6)		14 (10.9)		35 (19.3)	
Carcinoma in situ							
Yes	33 (8.2)	25 (9.1)	0.216	11 (8.6)	0.485	11 (6.1)	0.104
No	371 (91.8)	250 (90.9)		117 (91.4)		170 (93.9)	
Variant differentiation							
Negative	338 (83.7)	228 (82.9)	0.328	115 (89.8)	0.014	155 (85.6)	0.214
Positive	66 (16.3)	47 (17.1)		13 (10.2)		26 (14. 4)	
Recurrence							
Yes	150 (37.1)	103 (37.5)	0.467	49 (38.3)	0.413	74 (40.9)	0.103
No	254 (62.9)	172 (62.5)		79 (61.7)		107 (59.1)	
Cancer-specific death							
Yes	138 (34.2)	102 (37.1)	0.043	31 (24.2)	0.003	52 (28.7)	0.022
Other cause	53 (13.1)	34 (12.4)		17 (13.3)		24 (13.3)	
Alive	213 (52.7)	139 (50.5)		80 (62.5)		105 (58.0)	
Treatment							
TURBT ± mitomycin *	67 (16.6)	41 (14.9)		24 (18.8)		31 (17.1)	0.069
Intravesical BCG	186 (46.0)	119 (43.3)		71 (55.4)	0.007 ^b^	89 (49.2)	
Cystectomy	72 (17.8)	53 (19.3)		13 (10.2)	0.004 ^c^	26 (14.4)	
Chemo/radiotherapy	79 (19.6)	62 (22.5)	0.017 ^a^	20 (15.6)		35 (19.3)	

The χ2 test with Yates’ correction was performed; the value of *p* ≤ 0.05 was considered statistically significant; * transurethral resection with or without intravesical instillation of a single dose of mitomycin; ^a^ Chemo/radiotherapy vs other therapy modalities; ^b^ BCG (Bacillus Calmette–Guérin vaccine) intravesical vs other therapy modalities; ^c^ Radical cystectomy vs other therapy modalities.

**Table 2:** Multivariate analysis of the prognostic factors by Cox regression showing variables with influence on the overall survival of the patients.

	Overall Survival					
Parameter	B	SE	HR	95%CI		*p* Value
				Lower	Upper	
Tumor grade (high)	1.245	0.268	3.472	2.054	5.868	<0.001
Pathologic stage						
pT1	0.844	0.325	2.326	1.230	4.401	0.009
pT2	1.733	0.357	5.656	2.812	11.376	<0.001
Variant morphology (yes)	0.106	0.192	1.112	0.764	1.620	0.580
Carcinoma in situ (yes)	−0.163	0.242	0.850	0.529	1.365	0.501
TGF-β1 (high)	0.448	0.219	1.565	1.019	2.405	0.041
Smad2 (high)	0.052	0.204	1.053	0.705	1.572	0.800
Smad4 (high)	−0.140	0.174	0.869	0.619	1.222	0.420
